# 1327. The Diagnosis of Subarachnoid Neurocysticercosis Is Often Delayed And Other Findings of a Multicenter Retrospective in the USA

**DOI:** 10.1093/ofid/ofad500.1165

**Published:** 2023-11-27

**Authors:** Janitzio J Guzmán, Jesica A Herrick, Timothy Hatlen, Jill E Weatherhead, Eva Clark, Jose Serpa, Felicia Chow, Paul R Allyn, Noah Wald-Dickler, Jorge M Rodriguez, Natalie M Bowman, Anna Cervantes-Arslanian, Christina Coyle, A Clinton Clinton White, Senate Amusu, Dannae Martin, Megan M Duffey, Travis Larsen, Elliott M Welford, Jeffrey D Jenks, Anne Cowell, Andrés F Henao Martínez, Carlos Franco-Paredes, Glenn Mathisen, Paola Lichtenberger, Rory Bouzigard, Laila M Castellino, Elise M O’Connell

**Affiliations:** National Institute of Allergy and Infectious Diseases, Bethesda, Maryland; University of Illinois at Chicago, Chicago, Illinois; Harbor-UCLA Medical Center, Torrance, California; Baylor College of Medicine, Houston, Texas; Baylor College of Medicine, Houston, Texas; Baylor College of Medicine, Houston, Texas; University of California, San Francisco, San Francisco, California; UCLA, Los Angeles, California; LA County + University of Southern California Medical Center; University of Alabama at Birmingham, Birmingham, Alabama; University of North Carolina, Chapel Hill, North Carolina; Boston University and Boston Medical Center, Boston, Massachusetts; Albert Einstein College of Medicine, New York, NY; University of Texas Medical Branch, Galveston, TX; UIC, Chicago, Illinois; Lundquist Institute at Harbor-UCLA Medical Center, Torrance, California; Baylor College of Medicine, Houston, Texas; LAC + USC Medical Center, Los Angeles, California; UCSF, San Francisco, California; Durham County Department of Public Health, Durham, North Carolina; UC San Diego, La Jolla, California; University of Colorado Anschutz Medical Campus, Aurora, CO; Hospital Infantil de México, Federico Gómez, Denver, Colorado; Olive View-UCLA Medical Center, Sylmar, California; Infectious Disease Division. University of Miami, Miller School of Medicine., Miami, Florida; UT Southwestern, Dallas, Texas; University of Texas Southwestern Medical Center, Dallas, Texas; National Institute of Allergy and Infectious Diseases, Bethesda, Maryland

## Abstract

**Background:**

Subarachnoid (racemose) neurocysticercosis (SANCC) is an uncommon but severe form of *Taenia solium* infection. There is limited evidence to guide clinical management of these patients.

**Methods:**

We performed a multicenter retrospective chart review of 15 U.S. sites. A total of 69 subjects with racemose disease were entered.

**Results:**

The most common region of exposure was Mexico (67%) followed by Central America (24%). Median age was 43 years (range 15-76) and 71% were male. Common symptoms at the time of index admission were headache (80%), nausea/vomiting (46%), dizziness (44%), and blurry vision (33%). Cysts were intracranial in 64 (93%) subjects and exclusively intraspinal in 4. One patient had meningitis without visible cystic lesions. Incident admission magnetic resonance imaging (MRI) demonstrated ventriculomegaly in 41 (59%) and focal findings in 9 (13%) including ischemic infarct, subarachnoid hemorrhage, and/or arterial aneurysm. For 55 (80%), SANCC was first diagnosed during the index admission. Of these, 23 (42%) had prior medical visits and substantial delay in diagnosis (i.e. previously seen with hydrocephalus [27%], stroke [5.5%], and/or meningitis [11%], missed diagnostic radiologic features [4%], or inadequate imaging [5.5%]). Of the 69 subjects, 54% underwent a neurosurgical procedure during index admission (cyst removal n=16, EVD/shunt/ventriculostomy n=24). At the time of discharge, 6 (8.6%) patients were not given albendazole and/or praziquantel due to cost or availability. Six months following discharge, < 10% of follow up MRIs demonstrated cyst resolution. Planned treatment course of < 4 weeks at discharge compared to >4 weeks was associated with increased risk for new cyst development on follow up imaging at a median of 3.8 years following discharge (range 2.6 months-8 years). Those with a delayed diagnosis received a significantly longer duration of corticosteroids (median 8 weeks) than those without a delay (median 5 weeks, p=0.047).

**Conclusion:**

The diagnosis of SANCC is often missed, and most patients require neurosurgical intervention. Antiparasitic therapy is suboptimal, especially with regimens developed for parenchymal NCC.

**Disclosures:**

**Jeffrey D. Jenks, MD, MPH**, Astellas: Grant/Research Support|F2G: Grant/Research Support|Pfizer: Grant/Research Support

